# Light chain cast nephropathy caused by plasmablastic lymphoma of the bladder 

**DOI:** 10.5414/CNCS110339

**Published:** 2021-07-01

**Authors:** Mehrian Jafarizade, Kiran Goli, Vivette D’Agati, Essel Dulaimi, Krupa Daniel, Bradley Lash, Sharon Maynard

**Affiliations:** 1 Department of Medicine, Division of Nephrology, Lehigh Valley Health Network, Allentown, PA,; 2 Department of Pathology and Cell Biology, Columbia University Irving Medical Center, New York, NY,; 3 Department of Anatomic Pathology, Corporal Michael J. Crescenz VA Medical Center, Philadelphia, PA,; 4 Department of Geriatrics and Extended Care, VA Medical Center, Coatesville, PA, and; 5 Lehigh Valley Cancer Institute, Lehigh Valley Health Network, Allentown, PA, USA

**Keywords:** acute kidney injury, plasmablastic lymphoma, light chain cast nephropathy, HIV, lymphoma, bladder lymphoma, interstitial nephritis

## Abstract

Introduction: Plasmablastic lymphoma (PBL) is a rare form of B-cell lymphoma typically seen in patients with underlying immunosuppression such as HIV, autoimmune disease, and organ transplantation. PBL in HIV-positive patients usually originates from the gastrointestinal tract, with a predilection for the oral cavity. Bladder involvement by PBL is exceedingly rare, and cast nephropathy due to κ light chain-secreting PBL has not been reported previously. Case report: We report a patient who presented with acute kidney injury (AKI) in the setting of HIV, and was found to have a bladder tumor. Bladder pathology revealed a high-grade PBL with κ light chain restriction. Renal biopsy showed κ light chain cast nephropathy, presumably secondary to κ light chain-secreting PBL. Conclusion: Although the prognosis of PBL is poor, our patient recovered from AKI, achieved complete hematologic remission with chemotherapy, and underwent successful autologous stem cell transplant.

## Introduction 

Plasmablastic lymphoma (PBL) is a rare, aggressive form of B-cell lymphoma which was first described in 1997. Sixteen cases of highly malignant diffuse large B-cell lymphoma (DLBCL) were reported in the oral cavity with distinctive immunohistological features that distinguished it from other B-cell lymphomas, including negative immunoreactivity for CD20. Fifteen of these sixteen patients were HIV positive [1, 2]. In 2008, PBL was classified as a separate category by the World Health Organization (WHO) [3]. 

PBL was first described as an HIV-associated lymphoma. Shortly thereafter, cases of PBL were reported in the setting of other immunosuppressed states, such as autoimmune disorders and solid organ transplantation. In patients with HIV, PBL most often originates from the oral cavity. In case reports of PBL in non-HIV patients, PBL originates from other sites such as the retroperitoneum, gastrointestinal (GI) tract, and lungs [4, 5, 6, 7]. PBL involving the urinary tract is extremely unusual, and renal failure in association with PBL is rarely reported [8]. 

Kidney impairment in lymphoma can be directly related to malignancy such as obstruction of the ureters or renal vasculature by tumor or neoplastic infiltration of renal parenchyma. Alternatively, kidney injury can be caused by indirect effects of lymphoma such as hypercalcemia, paraproteinemia, immune-mediated glomerulonephritis (GN), and amyloidosis. Treatment-related complications include tumor lysis syndrome, chemotherapy-induced renal failure, and radiation nephritis [9]. In clinical practice, renal failure is often due to a combination of these factors. Prompt diagnosis and treatment are needed to prevent kidney damage. 

Herein, we present a case of acute kidney injury (AKI) due to light chain cast nephropathy (LCCN) associated with PBL of the bladder. LCCN is a rare complication of lymphoma, and this case represents the first clinical report of LCCN in PBL. 

## Case presentation 

A 39-year-old African American female with 20-year history of HIV infection and hypertension presented with complaints of abdominal pain and decreased urinary output of 2 weeks duration. She had sustained a mechanical fall 2 weeks prior to presentation and had taken ibuprofen for pain control. Initial evaluation revealed a serum creatinine 5.5 mg/dL. Her baseline serum creatinine 3 months prior to presentation was 0.91 mg/dL. Urinalysis showed 3+ proteinuria and microhematuria, without microscopic casts. Urine protein-to-creatinine ratio was > 10 g/g. CD4 count was 267/µL and HIV viral load by PCR revealed viremia with 1,220 copies/mL. She was compliant with antiretroviral therapy (ART) consisting of bictegravir, emtricitabine, and tenofovir alafenamide (Biktarvy), and lisinopril 10 mg daily for hypertension. 

Non-contrast computed tomography (CT) scan of the abdomen and pelvis showed mild bilateral hydronephrosis, marked diffuse irregular bladder wall thickening and ascites. Bladder cystoscopy revealed no evidence of ureteral obstruction but showed diffuse nodules throughout the bladder wall; multiple bladder biopsies were obtained. Given her renal insufficiency, tenofovir was stopped and ART was changed to renally adjusted dolutegravir, rilpivirine, and lamivudine. Serologic workup, including complement C3 and C4, antinuclear antibodies, hepatitis C antibody, hepatitis B surface antigen, anti-neutrophil cytoplasmic autoantibody (ANCA) titers, and anti-GBM antibody, were negative. Serum protein electrophoresis was consistent with acute inflammatory stress response, with no evidence for a monoclonal protein. However, serum free κ light chains were elevated at 1,988 mg/L, with serum free κ/λ ratio 36.4. Urine immunofixation revealed monoclonal free κ light chains. Attempted bone marrow biopsy was unsuccessful. Diagnostic paracentesis was negative for malignancy. Hemodialysis was initiated on hospital day 6 because of progressive renal failure and uremic symptoms. 

**Figure 1.**
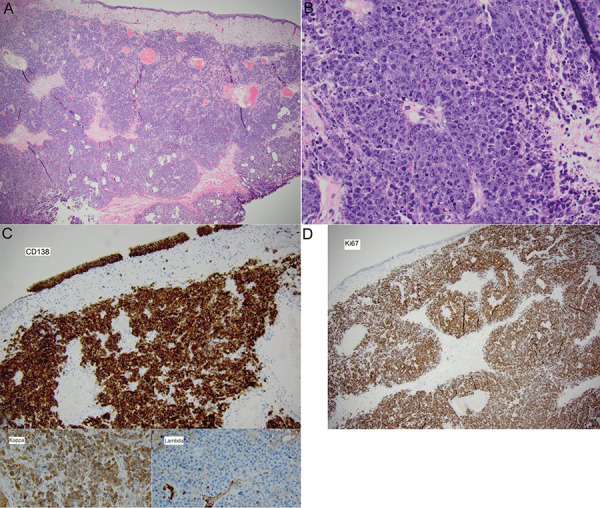
Plasmablastic lymphoma of the bladder. A: Transurethral bladder resection specimen. Intact urothelial mucosa with underlying hypercellular tumor infiltrating muscularis propria (H & E, × 10). B: Large plasmablastic cells with prominent eosinophilic nucleoli present in sheets, with frequent apoptotic bodies and mitotic figures (H & E, × 100). C: CD138 is expressed in lymphoma cells as well as the benign urothelial surface (× 10). Tumor cell stain diffusely positive for κ, negative for λ (inset, both × 100). D: Ki 67 demonstrated very high proliferation index approaching 100% (× 10).

Bladder biopsy revealed high-grade plasmablastic lymphoma (Figure 1). Histopathology showed large plasmablastic cells diffusely positive for CD138, MUM-1, and negative for PAX-5, CD20, BCL-6, and BCL-2. Cell proliferation marker Ki-67 approached 100%. Tumor cells were positive for EBER1. Additional immunostains revealed that the plasmablastic lymphoma cells were κ light chain-restricted and positive for CD56, CD10, and c-MYC. They were negative for λ light chain, CD30, and AE1/AE3. 

**Figure 2.**
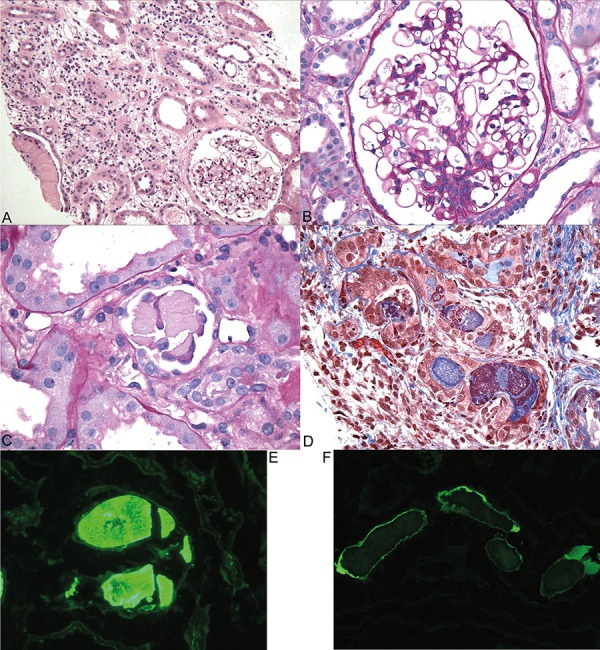
Kidney biopsy with focal atypical κ-restricted casts with acute tubular injury and patchy interstitial inflammation and edema, suggestive of κ light chain cast nephropathy. A: Low power view shows renal cortex with focal large fractured casts (far left on tissue edge) and interstitial expansion by edema and mononuclear leukocytes. A glomerulus appears unremarkable (H & E, × 100). B. A representative glomerulus is normal in size and cellularity, with no evidence of focal segmental sclerosis or collapsing glomerulopathy typical of HIV-associated nephropathy (Periodic-acid Schiff (PAS), × 600). C: A fractured PAS-negative cast with angulated contours and adherent mononuclear leukocytes is shown (PAS, × 600). D: Some of the atypical casts are polychromatic (staining trichrome-red and blue), associated with interstitial inflammation and edema. Tubular atrophy and interstitial fibrosis occupied ~ 10 – 15% of the cortex. (Masson trichrome, × 400). E: Immunofluorescence stain of a fractured crystalline cast is strongly positive for κ light chain (immunofluorescence, × 600). F: The casts stain negative for λ light chain (immunofluorescence, × 400).

Diagnostic kidney biopsy, performed 9 days after presentation, revealed focal atypical κ-restricted tubular casts with diffuse acute tubular injury and 30% interstitial inflammation and edema, consistent with κ light chain cast nephropathy (Figure 2). There was no evidence of glomerular disease by light microscopy, immunofluorescence, or electron microscopy. 

Chemotherapy was initiated 2 weeks after initial presentation with bortezomib and CHOP (cyclophosphamide, doxorubicin, vincristine, and prednisone). She received 1 dose of intrathecal methotrexate, cytarabine, and hydrocortisone followed by 3 doses of intrathecal methotrexate alone to prevent central nervous system (CNS) relapse, which is common in patients with HIV-associated lymphoma [10]. She was maintained on ART during this period and started on trimethoprim-sulfamethoxazole and acyclovir for opportunistic infection prophylaxis. 

Seven weeks after initial presentation, renal function recovered, and hemodialysis was discontinued. Eleven weeks after presentation, the patient achieved complete remission by imaging (PET). Her chemotherapeutic induction regimen was then switched to bortezomib with ifosfamide, carboplatin, and etoposide (ICE) as CHOP is considered insufficient treatment for PBL [11]. Six months after presentation, repeat bladder biopsy showed no evidence of PBL and additional testing including PET and bone marrow biopsy were negative confirming complete remission. Seven months after presentation, she underwent modified BEAM (BCNU, etoposide, cytarabine, melphalan) followed by autologous hematopoietic cell transplant. At last follow-up 8 months after autologous hematopoietic cell transplant, her renal function remains stable (creatinine 1.07 mg/dL) with minimal proteinuria (urine protein-to-creatinine ratio 0.36 g/g), normal plasma free light chains, and improved HIV viral load (364 copies/mL). She tolerated chemotherapy well, except for peripheral neuropathy and onychodystrophy which were attributed to her chemotherapy. [Table Table1]

## Discussion 

PBL is a rare, aggressive variant of DLBCL associated with HIV infection and immunosuppression. PBL accounts for less than 0.2% of all the lymphoid malignancies in the United States [12] and ~ 2% of HIV-associated lymphomas [13]. Strong male predominance of 4 : 1 has been noted in HIV-associated PBL, with lower ratio in non-HIV-associated PBL [11, 14, 15]. 

PBL typically contains immunoblastic or plasmablastic tumor cells with varying degrees of plasmacytic differentiation. Malignant cells have abundant cytoplasm with eccentric nuclei and prominent nucleoli. PBL cells are immunophenotypically distinct, expressing plasma cell markers including MUM1/IRF-4, CD38, and CD138 and lacking pan-B cell markers CD20 and CD79. Epstein-Barr virus (EBV)-encoded RNA (EBER1) is usually positive in PBL, as in this case [16]. Light chain restriction is a common finding in PBL [17]. In this case, the immunohistochemical pattern of 100% Ki-67 staining, and positivity for CD10, EBER, and c-MYC in the setting of HIV suggest this was PBL and not an extra-medullary plasmacytoma [18]. 

Although initial cases of PBL were predominantly reported in patients with HIV, subsequently PBL has been reported in recipients of organ transplants [11] and other immunosuppressed populations. [19] Solid organ transplant recipients are at increased risk for post-transplant PBL and EBV-related lymphoma secondary to chronic immunosuppressive therapy to prevent graft rejection. Post-transplant PBL is most often reported in kidney and heart transplant recipients [20]. 

Unlike diffuse large B-cell lymphoma, PBL usually presents in extra-nodal sites, most commonly the oral cavity, GI tract, and skin. Approximately 40% present with bone marrow involvement and B symptoms like fevers, weight loss, and night sweats [11]. Atypical extra nodal distribution of PBL can occur as orbital, adrenal, pleuropulmonary, paraspinal, and gonadal involvement [21, 22, 23]. PBL leading to hydronephrosis due to direct urinary tract involvement is exceedingly rare [8, 24, 25]. Only one prior case of bladder PBL has been reported to date [8]. The site of extra-medullary disease does not have a consistent impact on survival or prognosis, although disease limited to oral cavity may have a better prognosis [26]. Vigilance is required for the development of circulating monoclonal immunoglobulins, as occurred in this case, which can cause kidney injury. 

AKI in our patient was initially presumed to be secondary to obstructive uropathy. However, cystoscopy revealed no evidence of ureteral obstruction except for subtle calyceal fullness. The differential diagnosis for our patient’s AKI after the pathologic diagnosis of PBL had been established was broad. Our patient had been exposed to ibuprofen, a nonsteroidal anti-inflammatory (NSAID), prior to admission. NSAIDs predispose to ischemic acute tubular injury by inhibiting prostaglandin-mediated afferent vasodilation, reducing peritubular blood flow. NSAIDs can also cause acute interstitial nephritis and nephrotic-range proteinuria with minimal change disease [27]. Our patient had longstanding HIV treated with tenofovir alafenamide (TAF). TAF is a prodrug of tenofovir, a nucleotide reverse transcriptase inhibitor. Although TAF is less nephrotoxic than its predecessor prodrug tenofovir disoproxil fumarate (TDF), it can cause proximal tubule mitochondrial dysfunction and AKI [28]. Another diagnostic possibility in our patient was HIV-associated nephropathy (HIVAN) due to collapsing focal segmental glomerulosclerosis (FSGS), although this lesion has become less common in the era of effective antiviral therapies [29, 30]. The most common causes of kidney disease in patients with HIV on ART include immune complex glomerulonephritis (ICGN), diabetic nephropathy, tenofovir nephrotoxicity, and FSGS. HIVAN remains the leading cause of kidney disease in patients not on ART [31]. 

Finally, the presence of elevated free κ light chains in the urine raised the possibility of a kidney lesion related to her lymphoma. There are no published studies of kidney pathology in patients with plasmablastic lymphoma. However, kidney biopsy studies in patients with non-Hodgkin’s lymphoma have revealed various pathologies including lymphomatous infiltration, membranoproliferative GN, cryoglobulinemic GN, crescentic GN, membranous nephropathy, minimal change disease, immunotactoid GN, AL amyloidosis, cast nephropathy, light chain proximal tubulopathy, and intracapillary monoclonal deposits [32, 33]. In addition to establishing the diagnosis, kidney biopsy is helpful to determine prognosis and predict response to therapy; for example, the extent of cast formation and degree of interstitial fibrosis and tubular atrophy indicate prognosis in patients with light chain cast nephropathy [34]. The broad differential diagnosis in the setting of nephrotic-range proteinuria and worsening kidney function justified the decision to pursue kidney biopsy in our patient. 

Kidney biopsy in this case showed acute tubular injury, interstitial inflammation, and κ LCCN. AKI due to LCCN was initially described in patients with multiple myeloma, and later was described in patients with lymphoma [35]. LCCN is the most common tubulointerstitial lesion associated with circulating monoclonal light chains. The diagnostic histopathologic finding is the presence of angulated crystalline casts of monoclonal light chain within tubular lumina [36]. AKI occurs when immunoglobulin light chains in the tubular filtrate bind to uromodulin and produce casts, causing tubular obstruction and renal tubular epithelial cell injury [37]. Tubular degenerative changes are common in LCCN, resembling acute tubular necrosis. Interstitial edema and inflammation are also common features [38]. Our patient’s diffuse tubular injury may have been secondary to cast nephropathy, NSAID or tenofovir nephrotoxicity, or a combination of these factors [38]. Renal outcome in LCCN depends on age, initial glomerular filtration rate, baseline free light chain level and β2-microglobulin level, hematologic response to treatment, extent of cast formation, and degree of interstitial fibrosis/tubular atrophy [34]. 

In our patient, NSAID use, hyperuricemia, tenofovir exposure, and volume depletion may have contributed to the development of kidney injury. NSAIDs and volume depletion attenuate renal perfusion pressure and increase tubular protein concentration, affecting the interaction between uromodulin and light chains and increasing the risk of cast formation [35]. Other factors that may increase risk of cast nephropathy include high levels of urinary free light chains, iodinated contrast, hypercalcemia, and acute pyelonephritis [39]. 

Recently, monoclonal immunoglobulin-associated kidney diseases have become better defined. Monoclonal gammopathies are diagnosed when monoclonal immunoglobulin (MIg) is identified in the serum or urine resulting from a clonal proliferation of plasma cells or B lymphocytes. Most kidney diseases associated with MIg are a result of MIg or its light or heavy chain deposition in various compartments of the kidney. A variety of hematologic disorders are associated with MIg-kidney disease, including plasma cell dyscrasias and B-cell lymphoproliferative disorders. The term monoclonal gammopathy of renal significance (MGRS) is reserved for a non-malignant but clonal hematologic disorder that results in MIg-associated kidney disease. Clinical presentation can provide a clue to underlying diagnosis; however, kidney biopsy is required for definitive diagnosis [33]. 

While LCCN is a common cause of AKI in multiple myeloma [40], it should also be suspected in patients without multiple myeloma who have monoclonal protein detected in the urine or an abnormal serum free light chain ratio [41], as in our patient. We believe our patient’s bladder PBL was the source of LCCN because of κ-restricted staining of tubular casts on kidney biopsy, κ-restricted staining of bladder tumor cells, and the presence of κ free light chains in the urine. LCCN has rarely been reported in association with B-cell lymphomas [35, 42, 43]. To our knowledge, the current case is the first report of cast nephropathy caused by light chain-secreting PBL. 

We did not use extracorporeal methods (e.g., therapeutic plasma exchange) for light chain removal in our patient, instead opting for immediate chemotherapy. There is no data to guide the use of extracorporeal therapies for LCCN in patients with lymphoma. In patients with LCCN due to multiple myeloma, the use of extracorporeal therapies for removal of light chains is controversial, and a benefit has not been clearly established in clinical trials [44, 45, 46, 47]. If utilized, extracorporeal treatment must always be used along with appropriate chemotherapy which is essential in reducing the rate of light chain production. 

Prompt diagnosis and treatment to reduce serum free light chains are crucial for optimal renal recovery [40]. PBL generally carries a poor prognosis regardless of HIV status, with survival ranging from 7 to 11 months in different risk groups [11]. More recent data from the Lymphoma Study Association (LYSA) and the Surveillance, Epidemiology, and End Results (SEER) database showed better survival in patients treated with modern chemotherapy regimens, as used in our case [26, 48]. 

## Conclusion 

In summary, we report a case of severe AKI due to light chain cast nephropathy in the setting of plasmablastic lymphoma of the bladder in a patient with chronic HIV infection on ART. Kidney biopsy should be performed in patients with HIV and unexplained AKI for prompt diagnosis and appropriate treatment. Our patient achieved complete hematologic and renal remissions with bortezomib-based chemotherapy followed by autologous hematopoietic cell transplant. 

## Funding 

The authors received no specific funding for this work. 

## Conflict of interest 

None pertaining to this case report. 

**Table 1. Table1:** Laboratory parameters at diagnosis and follow-up.

Variable	On presentation	6 days after presentation (dialysis initiation)	6 months after presentation	Reference range
White blood cell count	10,700/mm^3^	8,700/mm^3^	6,500/mm^3^	4,000 – 10,000/mm^3^
Hemoglobin	10.2 g/dL	9.1 g/dL	12.1 g/dL	11.5 – 14.5 g/dL
Platelet count	470,000/mm^3^	489,000/mm^3^	336,000/mm^3^	140,000- 350,000/mm^3^
Blood urea nitrogen	27 mg/dL	42 mg/dL	19 mg/dL	7 – 25
Creatinine	5.55 mg/dL	11.36 mg/dL	1.05 mg/dL	0.60 – 1.20 mg/dL
HIV-1 RNA	–	1,220 copies/mL	1,030 copies/mL	< 20 copies/mL
CD4 T-cell count	–	267/µL	134/µL	401 – 1532/µL
Aspartate aminotransferase	39 U/L	–	15 U/L	13 – 39 U/L
Alanine transaminase	21 U/L	–	27 U/L	7 – 52 U/L
Total Bilirubin	0.3 mg/dL	–	0.4 mg/dL	0.2 – 1.1 mg/dL
Total protein	7.1 g/dL	–	9.0 g/dL	6.4 – 8.9 g/dL
Creatinine kinase	33 U/L		–	30 – 223 U/L
Uric acid	–	10.6 mg/dL	–	2.8 – 9.1 mg/dL
Lactate dehydrogenase	–	510 U/L	164 U/L	140 – 271 U/L
Spot urine protein to creatinine ratio	10 g/g		0.48 g/g	< 0.15 g/g
Serum Ig A	–	283 mg/dL	324 mg/dL	83 – 407 mg/dL
Serum Ig G	–	1109 mg/dL	1513 mg/dL	680 – 1,445 mg/dL
Serum Ig M	–	52 mg/dL	53 mg/dL	34 – 214 mg/dL
Serum protein electrophoresis	–	No monoclonal protein	No monoclonal protein	No monoclonal protein
Serum free light chains				
κ free light chains	–	1,888 mg/dL	42 mg/dL	3.30 – 19.40 mg/dL
λ free light chains	–	55 mg/dL	25 mg/dL	5.7 – 26.30 mg/dL
κ : λ free light chain ratio	–	36.43	1.69	0.26 – 1.65
Urine immunofixation	–	Positive for monoclonal κ free light chain	–	negative
